# Environmental selection of protistan plankton communities in hypersaline anoxic deep-sea basins, Eastern Mediterranean Sea

**DOI:** 10.1002/mbo3.56

**Published:** 2012-12-13

**Authors:** Sabine Filker, Alexandra Stock, Hans-Werner Breiner, Virginia Edgcomb, William Orsi, Michail M Yakimov, Thorsten Stoeck

**Affiliations:** 1 School of Biology, University of KaiserslauternErwin-Schroedinger-Str. 14, D-67663, Kaiserslautern, Germany; 2 Department of Geology and Geophysics, Woods Hole Oceanographic InstitutionWoods Hole, Massachusetts, 02543; 3 Institute for Coastal Marine Environment, IAMC-CNRSpianata S. Raineri, 86, 98122, Messina, Italy

**Keywords:** DHAB, environmental selection, protists, T-RFLP

## Abstract

High salt concentrations, absence of light, anoxia, and high hydrostatic pressure make deep hypersaline anoxic basins (DHABs) in the Eastern Mediterranean Sea one of the most polyextreme habitats on Earth. Taking advantage of the unique chemical characteristics of these basins, we tested the effect of environmental selection and geographic distance on the structure of protistan communities. Terminal restriction fragment length polymorphism (T-RFLP) analyses were performed on water samples from the brines and seawater/brine interfaces of five basins: Discovery, Urania, Thetis, Tyro, and Medee. Using statistical analyses, we calculated the partitioning of diversity among the ten individual terminal restriction fragment (T-RF) profiles, based on peak abundance and peak incidence. While a significant distance effect on spatial protistan patterns was not detected, hydrochemical gradients emerged as strong dispersal barriers that likely lead to environmental selection in the DHAB protistan plankton communities. We identified sodium, magnesium, sulfate, and oxygen playing in concerto as dominant environmental drivers for the structuring of protistan plankton communities in the Eastern Mediterranean DHABs.

## Introduction

The dissolution of outcropping ancient subterranean salt deposits from the Messinian salinity crisis (late Miocene period, >5 million years ago, Camerlenghi 1990) gave rise to a number of deep hypersaline anoxic basins (DHABs) in the Eastern Mediterranean Sea. These basins are located at a depth of more than 3000 m below sea level. Due to the high densities of the brines (up to 1.23 kg m^−3^, La Cono et al. 2011), mixing of these water masses with overlying deep-sea water (average density: 1.03 kg m^−3^) is restricted, resulting in anoxic conditions in these brines. In each basin, a halocline (typically 1–3 m deep) separates the anoxic brine from the normoxic and normsaline deep-sea water. High salt concentrations up to saturation, high pressure exceeding 300 atm, and a lack of oxygen make these polyextreme basins some of the harshest environments on our planet, challenging our notions of the limits of life. Despite these polyextreme conditions, bacteria and archaea flourish in the haloclines and brines of these deep-sea basins (Sass et al. 2001; van der Wielen et al. 2005; Daffonchio et al. 2006; Yakimov et al. 2006; van der Wielen and Heijs 2007). The haloclines in particular contain elevated microbial activity. Chemoautotrophic microbes in the haloclines receive reduced compounds from the hypersaline brines, which they can oxidize to exploit the available nutrient sources (Yakimov et al. 2007). Recent studies also provided evidence for the existence of diverse assemblages of polyextremophile eukaryotic life in some of the Eastern Mediterranean deep brine basins (Alexander et al. 2009; Edgcomb et al. 2009, 2011b; Stock et al. 2012). With the exception of three reported species of Loricifera thought to spend their entire life cycle in DHAB sediments (Danovaro et al. 2010), the eukaryotic life in the DHABs is predominantly microbial. The prokaryote network in these habitats most likely supports this unicellular protistan plankton, primarily consisting predominantly of alveolates, stramenopiles, and kinetoplastids (Alexander et al. 2009; Edgcomb et al. 2009, 2011b; Stock et al. 2012).

Most basins, including Thetis, Urania, Tyro, Bannock, and L'Atalante are thalassohaline, meaning that they originated from the evaporation of seawater. As a result, the proportion of major ions in thalassohaline environments is usually similar to seawater. However, due to the dissolution of different strata of evaporites from the Messinian salinity crisis, the hydrochemistries of Eastern Mediterranean Sea DHABs differ significantly. For example, salinity in Thetis, L'Atalante, Bannock, and Tyro ranges between 321 and 352 g/L (nearly 10 times higher than average seawater salinity), whereas Urania brine exhibits a salinity of only 240 g/L. Potassium ions range between 19 (Tyro) and 300 mmol/L (L'Atalante), sulfate between 52 (Tyro) and 323 (L'Atalante) mmol/L, sulfide between 2.1 (Thetis and Tyro) and 15 (Urania) mmol/L (La Cono et al. 2011), and methane between 0.4 (Bannock) and 5.6 (Urania) mmol/L (van der Wielen et al. 2005). In contrast, the Discovery brine is athalassohaline with its ionic composition differing greatly from thalassohaline brines. The most striking difference is the presence of monovalent cations in the thalassohaline brines (Na^+^ and K^+^), whereas the Discovery brine is characterized by divalent cations (Mg^2+^) and very low concentrations of Na^+^. The MgCl_2_ concentration in the Discovery basin (5 mol/L, van der Wielen et al. 2005) is the highest reported thus far in any marine environment and was previously considered anathema to life and a biogeochemical dead end (Horowitz et al. 1972; Siegel et al. 1979; Coleman 1993; Oren 1999). Monovalent and divalent cation salts have different effects on a cellular level (Hofmeister ion effects). While sodium salts decrease the solubility of proteins in a cell (salting-out effect), magnesium salts destabilize proteins by increasing their solubility (unfolding of proteins, salting-in effect) (Kunte et al. 2002; Stock et al. 2012). The cellular mechanisms to cope with magnesium salts are still unknown.

Because of their unique hydrochemistries and physical separation, the DHABs have the potential to serve as island habitats. This presents an opportunity to investigate physical and geochemical factors responsible for shaping protistan biogeography in these environments. We assessed the influence of physical separation and hydrochemistry on protistan brine communities by analyzing molecular fingerprint patterns of protistan communities in the brines and interfaces of Discovery, Urania, Thetis, Tyro, and Medee basin using terminal restriction fragment length polymorphism analysis (T-RFLP). Other studies have reported significant differences in protist communities between DHAB habitats (Alexander et al. 2009; Edgcomb et al. 2009, 2011b; Stock et al. 2012). This is the first study, however, to examine whether unique communities will be found across a broad comparison of DHAB brines and interfaces, and to specifically test whether distance between basins and basin hydrochemistry are primary drivers of any observed differences. While T-RFLP has the potential to underestimate diversity, and does not provide information on specific taxonomic groups, it is a powerful approach for our objective, which was to make comparisons of DHAB protistan community composition across many samples representing different hydrochemistries, and separated from one another by a range of distances. Even though distance-decay effects on spatial distribution patterns in microbial community structures, including protists and fungi, have been reported previously (e.g., Hillebrand et al. 2001; Green et al. 2004; Martiny et al. 2006 and references within; Wetzel et al. 2012), we hypothesize that distance effects do not play a pivotal role in structuring DHAB communities. This is because it is reasonable to assume that the protistan biota occurring in the hypersaline basins must have been recruited from the surrounding seawater or sediments underlying aerobic normsaline water.

## Materials and Methods

### Sampling sites and sampling

**Figure 1:**
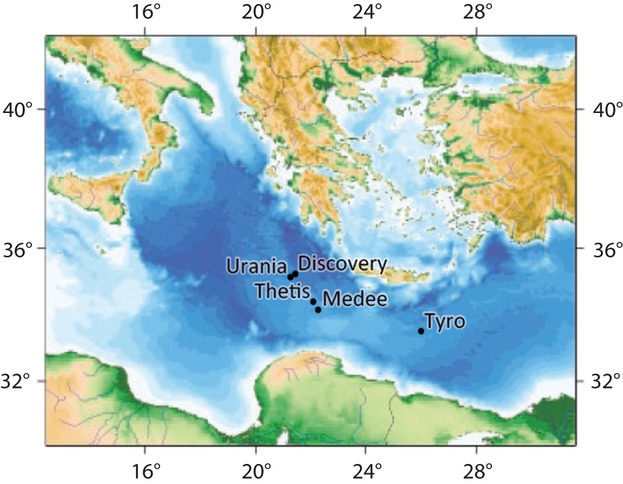
Map of deep hypersaline anoxic basins (DHABs) sampled in this study.

Samples were collected during two expeditions, including a cruise to Discovery and Urania basins in July 2009 on the R/V *Oceanus,* and in September 2009 on the R/V *Urania* (MIDDLE09 cruise) (Fig. 1). The position of the halocline was determined during the R/V *Oceanus* and *Urania* cruises using a SBE911*plus* CTD (Sea-Bird Electronics, Bellevue, WA) equipped with an SBE43 oxygen sensor (Sea-Bird Electronics). Samples were collected from the halocline and brine of each basin using a rosette equipped with 12-L Niskin bottles. The use of Niskin bottles for this study allowed us to capture approximately half the thickness of the halocline at each basin, starting where salinity no longer reflected normal seawater. Table 1 presents coordinates, depth, and hydrochemistry data for each sampling location/depth. The salinity gradient from the top to the bottom of individual Niskin bottles was confirmed on board the ship using a WTW portable sensor for conductivity, pH, and temperature (WTW, Weinheim, Germany). Water samples were collected from Niskin bottles into 50-L Nalgene bottles flushed with argon gas and filtered immediately onto Durapore membranes (47 mm; 0.65 μm; Millipore, Schwalbach am Taunus, Germany) under gentle vacuum (flow rate: ca. 50 mL/min) and under argon in the case of anoxic samples (Alexander et al. 2009), followed by storage in RNA*later* (Ambion, Applied Biosystems, Darmstadt, Germany). According to Ambion's RNA*later* manual, the filters were stored at 4°C for 24 h prior to freezing at −20°C until RNA extraction. Further physicochemical parameters (Table 1) were determined as described in (La Cono et al. 2011).

**Table 1:** Coordinates, sampling depths, and physicochemical data of the brines (B) and halocline interfaces (I) of the different DHABs under study

	Coordinates (Long, Lat)	Depth (m)	Salinity^1^ (PSU)	Conductivity^1^ (S/m)	Oxygen^1^ (mL/L)	Na^+^ (mmol)	Mg^2+^ (mmol)	SO_4_^2−^ (mmol)	HS^−^ (mmol)
DB	21.412304 E, 35.163666 N	3581	95	11.3	0	68^2^	4995^2^	96^2^	0.7^2^
MB	22.312124 E, 34.19468 N	2950	320	16.7	0	4818	792	201	2.9
TB	26.21962 E, 33.524236 N	3448	321	16.7	0	5300^3^	71^3^	53^3^	2.1^3^
ThB	22.084368 E, 34.401134 N	3380	348	16.7	0	4760^3^	604^3^	265^3^	2.1^3^
UB	21.283252 E, 35.13528 N	3493	240	15.6	0	3505^3^	315^3^	107^3^	15
DI	21.412304 E, 35.163666 N	3579	38	7.1	0.5	27.2	1998	NA	NA
MI	22.312124 E, 34.19468 N	2924	70	7.7	0.5	847	161	41	NA
ThI	22.084368 E, 34.401134 N	3259	80	8.2	0.68	1368	174	76	0.11
TI	26.21962 E, 33.524236 N	3327	67	7.8	0.5	1111	15	11	0.07
UI	21.283252 E, 35.13528 N	3468	63	7.8	1.22	876	79	42	0.66

D, Discovery; M, Medee; T, Tyro; Th, Thetis; U, Urania; NA, not available. Data are from the literature and from this study (measured as described in La Cono et al. 2011).

1 From Edgcomb et al. (2011b).

2 From van der Wielen et al. (2005).

3 From La Cono et al. (2011).

### envRNA extraction and transcription

The method for the extraction and transcription of environmental RNA (envRNA) from protistan plankton collected on membranes has been described in detail previously (Alexander et al. 2009). In short, total RNA was extracted using Qiagen's AllPrep DNA/RNA Mini kit (Qiagen, Hilden, Germany) according to the manufacturer's instructions following a chemo-mechanical cell disruption by bead-beating (45 sec, 30 Hz). Residual DNA was removed by DNase I (Qiagen) digestion. The concentration of extracted and purified RNA was determined spectrophotometrically using a Nanodrop ND-1000 UV-Vis spectrophotometer (Nanodrop Technologies, Wilmington, DE). The integrity of the RNA was checked with an RNA 6000 picoassay using an Agilent 2100 Bioanalyzer (Agilent Technologies, Germany). To minimize extraction bias, total RNA from three individual filters per depth and sampling site were extracted. Total RNA was then transcribed into cDNA using Qiagen's QuantiTect Reverse Transcription kit using primer Euk1179R (Hendriks et al. 1989, 5′-CCC GTG TTG ACA AA-3′) according to the manufacturer's instructions. After transcription of each individual sample, the transcribed products of each depth/sampling site were pooled and subjected to small ribosomal subunit (SSU) cDNA amplification.

### Oligonucleotide primers and polymerase chain reaction amplification

To amplify the SSU rRNA genes of the 10 individual microbial communities from the DHAB interfaces and brines of the five basins in this study, we conducted a semi-nested polymerase chain reaction (PCR), with the following universal eukaryote primer sets. For the first reaction, EukA (Medlin et al. 1988, 5′-AAC CTG GTT GAT CCT GCC AGT-3′) was used as a forward primer and Euk1179R as the reverse primer. In the second reaction, EukA served as the forward primer and Euk516R (Wuyts et al. 2004, 5′-ACC AGA CTT GCC CTC C-3′) as the reverse primer. In the second reaction, EukA was labeled with the phosphoramidite dye 6-carboxyfluorescein (6-FAM) at the 5′-end.

The PCR mixtures contained 30–90 ng of template cDNA, 5 U of HotStar *Taq* DNA polymerase (Qiagen, Hilden, Germany), 1× CoralLoad PCR Buffer (containing 1.5 mmol/L MgCl_2_), 200 μmol/L concentrations of each deoxynucleotide triphosphate, and 0.25 μmol/L concentrations of each oligonucleotide primer. The final volume was adjusted to 50 μL with sterile water. The PCR protocol for both reactions, amplifying ca. 1100-bp-long SSU rRNA gene fragments in the first reaction and ca. 500-bp fragments in the second reaction, consisted of an initial denaturation (5 min at 95°C) followed by 28 identical amplification cycles (denaturation at 95°C for 30 sec, annealing at 58°C for 30 sec, and extension at 72°C for 1 min), and a final extension at 72°C for 5 min. Finally, PCR results were checked by agarose gel electrophoresis (1%), and after purification (MinElute PCR Purification Kit, Qiagen, Hilden, Germany) the DNA yield was quantified photometrically with the NanoDrop 2000 Spectrophotometer of NanodropTechnologies (Wilmington, DE). In order to minimize PCR-related bias, each extracted template DNA was amplified in six individual PCR reactions. First-reaction amplicons were pooled (per template) after MinElute-purification and before second PCR. After the second PCR (again six individual reactions per template), replicates were not pooled, but digested individually with restriction endonucleases.

### Restriction digestion of cDNA amplicons

To minimize the risk of the formation of artificial terminal restriction fragments (pseudo-T-RFs) due to inefficient restriction digestion of single-stranded PCR amplicons, we first digested PCR products with a single-strand-specific mung bean nuclease (Egert and Friedrich 2003). PCR products were incubated with 10× mung bean nuclease digestion reaction buffer and 1 μL of the enzyme (10 units/μL, New England Biolabs, Ipswich, MA) at 30°C for 30 min. Addition of sodium dodecyl sulfate (SDS) (0.01% final) terminated the reaction. Purification of the PCR products was carried out by isopropanol precipitation. The DNA yield was quantified photometrically using the NanoDrop 2000 Spectrophotometer of Nanodrop Technologies (Wilmington, DE).

An important step for T-RFLP analyses is the choice of restriction enzymes. As the goal of our analyses was the detection of diversity and differences in community structure, we chose enzymes that produce a large number of T-RFs with a reasonable length distribution. On the basis of literature data, we chose three restriction endonucleases for digestion of the amplification products, which are routinely used in protistan T-RFLP fingerprinting (Massana and Jürgens 2003; Engel et al. 2011): *Hae*III, *Hha*I, and *Msp*I (all from New England Biolabs). Restriction digestion was carried out in 10-μL reactions according to the manufacturer's instructions using 5 μL of each purified PCR product (ca. 0.3–1.5 μg DNA). Digestion products were desalted by isopropanol precipitation and resuspended in 10 μL of deionized sterile water. Each restriction digestion for each enzyme and sample was performed in six replicates, each replicate per enzyme originating from a separate PCR reaction. Prior to the analysis of T-RFLP reactions using a 3730 DNA analyzer (Applied Biosystems, Carlsbad, CA) at Seq-It laboratories (Seq-It GmbH, Kaiserslautern, Germany), 15 ng of purified digestion products were each mixed with 7.5 μL of a HiDi formamide solution (Applied Biosystems) and 0.5 μL of a ROX-labeled MapMarker1000 (Eurogentec, Cologne, Germany).

### Processing of T-RFLP electropherograms

Electropherograms derived from the T-RFLP runs were aligned into bins and analyzed using GelQuest^©^ (version 2.1.2.SequentiX-digital DNA processing, Klein Raden, Germany) using default settings except for the following parameters: smoothing width, 10; baselining width, 50; minimum peak height for T-RFs, 75; minimum peak height for marker, 200; and hyperbin width, 1.0. Finally, to distinguish signal from noise using a constant percentage threshold, only those T-RFs were taken into account that contributed at least 1% to the relative fluorescence (*r*A, based on height of T-RFs) of a sample (Noll et al. 2005). The relative abundance (*r*A) of each T-RF was calculated as *r*A = *n*_*i*_ × 100*/N,* in which *n*_*i*_ represents the peak height of one distinct T-RF and *N* is the sum of all peak heights in a given T-RFLP profile. *r*A values were determined for all T-RFs detected in a size range between 50 and 700 bp for a given T-RFLP profile. Finally, data from six replicate reactions per sample were assembled by calculating the average r*A* of each individual T-RF to generate consensus T-RFLP profiles (one for each sample).

### Statistical analyses of T-RFLP profiles

Similarities between communities were calculated with two different indices: (i) the Jaccard index, which is based on the presence/absence of a T-RF (binary variables of peak presence). This coefficient is equal to the ratio of matching T-RFs in two profiles and the total number of T-RFs present in either profile (Legendre and Legendre 1998); (ii) an abundance-based modification of the Sørensen Index (Chao–Sørensen), which takes into account relative fluorescence units as quantitative data (Chao et al. 2005). Both indices were calculated using the software EstimateS v.8 (Colwell 2009), and then translated into distance matrixes (1 minus Jaccard or Chao–Sørensen index value) for UPGMA cluster analyses.

To assess an effect of distance on community similarities, Jaccard and Chao–Sørensen indices were plotted against distance data among individual sample sites in a Pearson rank correlation using the Statistica software package. A Student's *t*-test for paired samples was used for significance testing. Geographic distances were calculated via the subtraction of different depths on a single geographic position, which resulted in the altitude difference within the same basin. For the calculation of the two-dimensional great-circle distance between two points on a sphere from their longitudes and latitudes (same depth), the haversine formula (Sinnott 1984) was implemented in the script as provided by Chris Veness (2002–2011) at http://www.movable-type.co.uk/scripts/latlong.html.

A canonical correspondence analysis (CCA) of T-RFLP profiles (including T-RF size and relative abundance data) was conducted to describe the relationships between community composition patterns and underlying environmental gradients, which shape these diversity patterns. Data were log-transformed (Grant and Ogilvie 2003) and unconstrained permutations (*n* = 499) were run under a reduced model. Monte Carlo significance tests of first ordination axes and of all canonical axes together were performed. Initially, all available environmental variables (see above) were included in the model. In order to develop a robust model explaining as much variance as possible while avoiding multicollinearity, individual variables were removed in a step-wise manner. We used the Canoco software (Microcomputer Power, Ithaca, NY) for the ordination analysis.

A presence/absence map, visualizing the occurrence of specific T-RFs in each individual sample, was generated using the tool Heatmap Builder (King et al. 2005).

## Results

### Similarities of site-specific T-RFLP profiles

**Figure 2:**
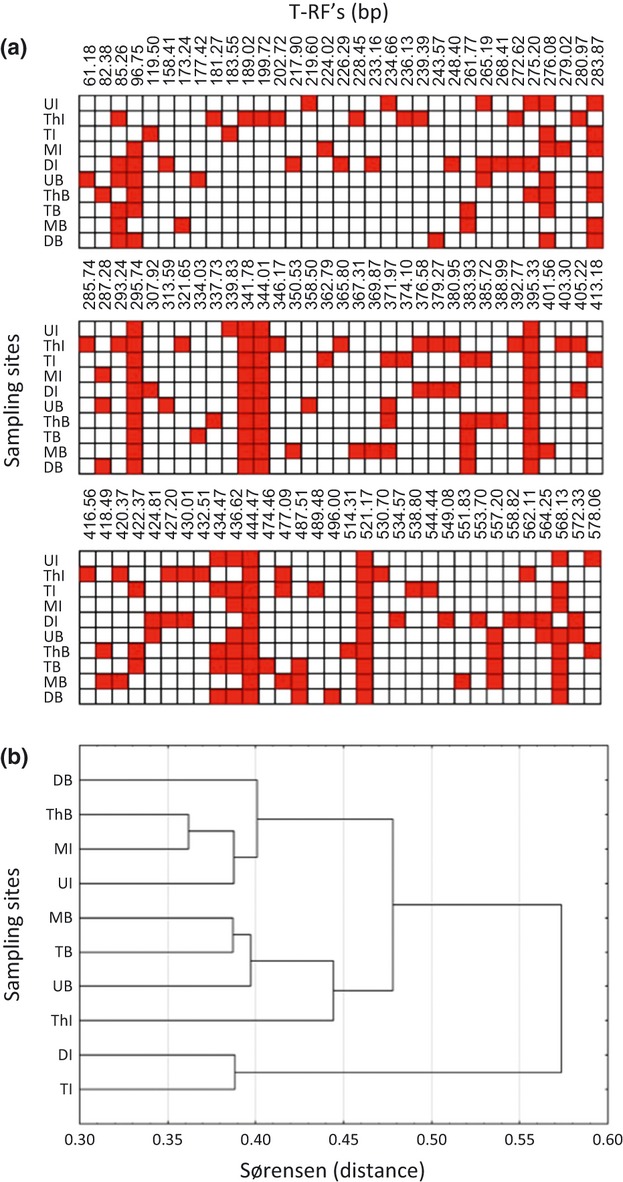
(a) Presence/absence map of terminal restriction fragments (T-RFs) obtained from three different restriction enzymes. Colored boxes indicate the presence of a specific T-RF at brines (B) and halocline interfaces (I) of the different sampling sites. (b) Hierarchical clustering (Sørensen distance) of sampling sites based on presence/absence of T-RFs. D, Discovery; M, Medee; T, Tyro; Th, Thetis; U, Urania.

The number of T-RFs obtained from each habitat ranged between 17 (Discovery brine) and 55 (Tyro halocline) (Table 2, Fig. 2a). Cluster analysis of the abundance-based Chao–Sørensen index identified three clusters (Fig. 2b). Identical clusters were also recovered with the incidence-based Jaccard index cluster analysis (Fig. S1). The communities of Medee, Tyro, and Urania brines are more similar to each other than to those of Discovery and Thetis brines. The brine communities are distinct from those detected in the halocline samples. None of the halocline T-RFLP profiles show high degrees of similarity to each other.

**Table 2:** Number of eukaryote-specific T-RFs as a measure of α-diversity in each of the habitats profiled with T-RFLP

	Discovery		Medee		Thetis		Tyro		Urania	
					
SITE	B	I	B	I	B	I	B	I	B	I
# T-RFs	17	44	32	22	25	27	30	50	37	19

B, brine; I, halocline interface.

### Effect of environmental variables on eukaryote community profiles

**Figure 3:**
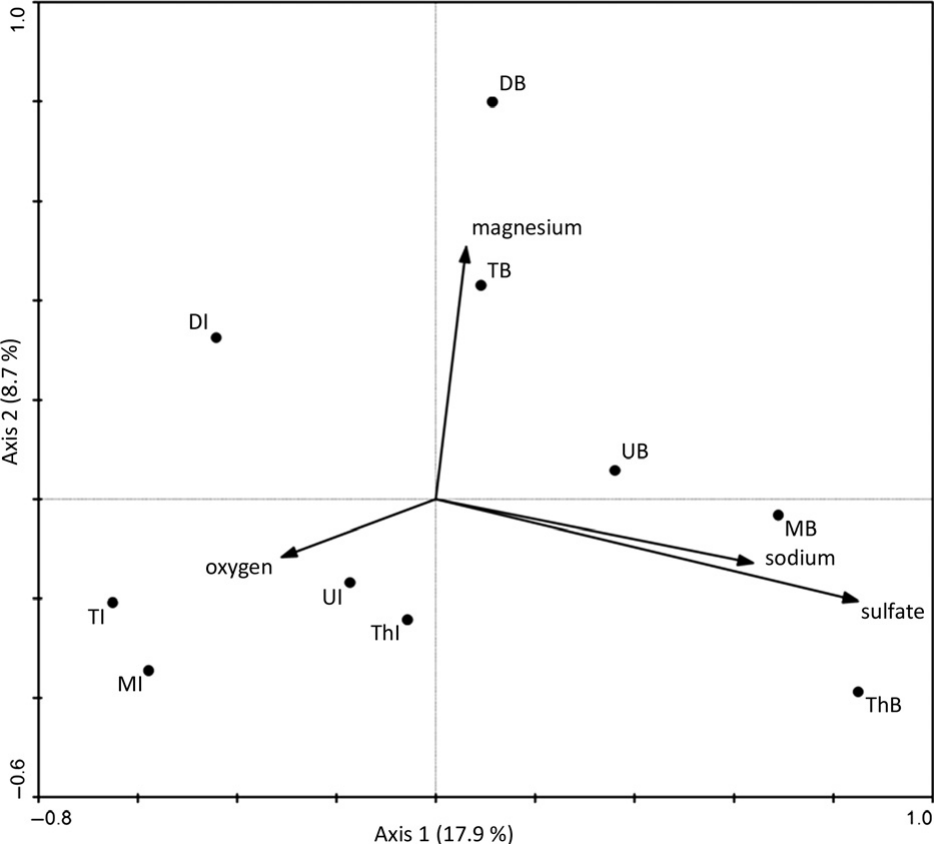
Canonical correspondence analysis (CCA) of T-RFLP profiles (including T-RF size and relative abundances) for brines (B) and halocline interfaces (I) of the different sampling sites. This CCA depicts the best model in our CCAs, explaining 38% of the total variation within the T-RFLP profiles with the first two axes accounting for 27%. The first two canonical axes explained 70% of the variation of the species–environment relation. Magnesium concentration is significantly (positively) correlated with the first axis (*P* = 0.05). D, Discovery; M, Medee; T, Tyro; Th, Thetis; U, Urania.

The resulting ordination diagram of the CCA expresses not only the pattern of variation between T-RFLPs, but the main association between the observed T-RFs and each of the environmental variables. Environmental variables with arrows close to the canonical axes may explain a large proportion of the variation accounted for by this axis. The longer the arrow, the more variation may be explained by this factor. The best model in our CCA explained 38% of the total variation within the T-RFLP profiles with the first two axes accounting for 27% (Fig. 3) and the first two canonical axes explaining 70% of the variation of the species–environment relation. Magnesium concentration is significantly correlated with the second axis (*P* = 0.05). Salinity was significantly correlated to the first axis (*P* < 0.05) when included as an additional environmental variable. However, because (i) sodium concentration and salinity show a collinearity and (ii) the first two axes explain only 68% of the variance of species–environment relations, the model without salinity data was preferred.

The availability of oxygen as well as the concentrations of sodium and sulfate unite all halocline samples in the ordination diagram. The brine samples cluster separately from halocline samples in the direction of the sulfate, magnesium, and sodium vectors. Thus, high magnesium and lower sulfate and sodium concentrations are in part responsible for the pronounced structural differences of the Discovery brine eukaryote community compared with the protistan plankton communities of Tyro, Urania, Medee, and Thetis brines as the communities and sample sites are arranged on a nearly linear gradient along these variables (Fig. 3).

### Distance effect on DHAB eukaryote community profiles

Distance dependence was low, and very little of the overall variability in community similarities was accounted for by the regression model (*R*^2^ = 0.08). A correlation between distance and community similarity is insignificant (*P* = 0.16).

## Discussion

T-RFLP is a commonly used and efficient molecular fingerprinting technique to monitor changes and differences in the structure and composition of microbial populations in various habitats (Countway et al. 2005; Noll et al. 2005; Murase et al. 2006; Mou et al. 2007; Joo et al. 2010). This method was introduced about 15 years ago (Liu et al. 1997) and since then a wide range of statistical techniques have been adopted and used to assess and compare T-RFLP profiles. We used the T-RF approach here to investigate the influence of DHAB hydrochemistry on selection of protistan communities on a broad scale. Therefore, we analyzed samples collected in July and September 2009. In order to assess the effect of different sampling times, we compared the T-RFLP-patterns of one basin (Thetis) that was sampled at both points in time. We found that the temporal variation in Thetis between July and September samples is negligible (insignificant chi-square test) in comparison with the spatial variation observed between different basins. The complete T-RFLP profiles are available from the authors upon request.

It is important to choose appropriate statistical strategies when addressing hypotheses using T-RF data. For instance, it is important to decide whether incidence-based (community composition) or abundance-based (community structure) T-RF data should be used as distance measures. Additionally, it is important to decide whether the absence of a peak in two samples should be regarded as a similarity between these two samples or whether such an observation should have no impact on the distance between two profiles (Schütte et al. 2008). These choices may bias the interpretation of the results.

In order to assess beta-diversity across biogeographic barriers (between different isolated DHABs) and along hydrochemical gradients (interfaces), we calculated the similarities between the ten individual T-RF profiles based on both peak abundance and peak incidence. The Jaccard index, which we used to assess community composition, only accounts for T-RF presence, not absence. It was suggested that the absence of a microbial population in one of two profiles should not impact the similarity between these profiles, because the failure to detect a population in one profile may not mean that it is truly absent from a sample, but instead it could be below the detection limit (Schütte et al. 2008). Interestingly, our cluster analyses of community compositions (Fig. S1) recovered the same clusters as the community membership analyses (Fig. 2b). These results suggest that the geochemical gradients within the haloclines act as biogeographic barriers. This finding is in line with a recent study of protistan community structure in the Cariaco Basin, which also found that geochemical gradients are biogeographic barriers to protistan dispersal (Orsi et al. 2011). This restriction of protists to different DHABs likely results in selection of unique protistan communities adapted to the specific conditions of each basin. The observation of unique T-RF profiles in the majority of samples supports this hypothesis (Fig. 2).

Several molecular studies, including fingerprinting profiles, have reported a significant correlation between changes in microbial community structure/composition and linear distance ranging from large scales (up to 20,000 km; Cho and Tiedje 2000) to small scales (200–0.01 km; Oda et al. 2003; Sliwinski and Goodman 2004). However, it remains unresolved how much of the observed spatial variation in microbial communities is due to environmental conditions or to distance (Martiny et al. 2006). One study (Martiny et al. 2006) concluded that at intermediate scales (3–10,000 km) biogeographic provincialism may be an effect of both environmental conditions and distance.

We were not able to detect a significant distance effect for the five brine and the five interface protistan plankton communities. Instead, our data suggest that the unique geochemical gradients within the different DHABs are responsible for the observed dissimilarities among the protistan communities (Fig. 3). In contrast to principal component analyses (PCA) and nonmetric multidimensional scaling (MDS, NMS, NMDS), constraint ordinations such as CCAs link community changes to environmental differences (Ter Braak 1986). The CCA identified salinity, sulfate, magnesium, and oxygen as important environmental parameters driving the selection of the sampled protistan communities.

Salinity shifts characterize a boundary, which is one of the most difficult barriers to cross for organisms from all three domains of life. Lozupone and Knight (2007) analyzed global bacterial distribution patterns along a salt gradient from freshwater to marine and found that the major environmental determinant of bacterial community composition is salinity. Likewise, Logares et al. (2009) provide evidence that transitions across salinity gradients are rarely made in the microbial world, including by eukaryotic microbes. Examples of salinity gradient segregation in unicellular eukaryotes include dinoflagellates, diatoms, chlorophytes, cryptomonads, foraminifera, heliozoans, goniomonads, choanoflagellates, bicosoecids, placidomonads, and a number of diverse heterotrophic flagellates (see references in review by Logares et al. 2009). Also, numerous ciliates show specific distribution patterns along salinity gradients (Urrutxurtu et al. 2003; Mazei and Burkowski 2006; Lei et al. 2009). Several of the protistan lineages mentioned above were detected previously in initial molecular diversity surveys in some of the Eastern Mediterranean Sea DHABs (Alexander et al. 2009; Edgcomb et al. 2009, 2011b; Stock et al. 2012). Specifically, diverse populations of alveolates (dinoflagellates, ciliates) have been detected within the DHABs. The energetic costs of osmoregulation as well as the evolution of adaptations to high salt concentrations may be among the most important factors limiting the distribution of organisms along salt gradients (Oren 2001, 2002). Furthermore, as shown for animals (Vermeij and Dudely 2000), the success of transitions among physically different environments such as in salt gradients may be diminished through the competition of locally well-adapted taxa, preventing invaders from establishing large populations.

Like salt, the concentration of oxygen may be an environmental barrier, which is difficult to overcome for most organisms, including protists (Fenchel and Finlay 1995). Many protists have adapted an anaerobic lifestyle several times during their evolutionary history (Mentel and Martin 2010). For example, some ciliates possess hydrogenosomes, modified mitochondria that anaerobically produce hydrogen and ATP, that have multiple independent origins in the evolution of ciliates (Embley et al. 1995; Biagini et al. 1997; Hackstein et al. 2001). Also anaerobic mitochondria, which may represent transition states between aerobic mitochondria and hydrogenosomes are widespread among ciliates, foraminifera, and euglenids that are found in oxygen-depleted habitats (Fenchel and Finlay 1995). For protists with such anaerobic organelles, oxygen can pose an environmental barrier. Previous molecular diversity surveys of protists in aquatic habitats demonstrated that an oxygen gradient selects for specifically adapted taxa (Stoeck and Epstein 2003; Stoeck et al. 2003, 2009; Behnke et al. 2006, 2010; Alexander et al. 2009; Edgcomb et al. 2009; Orsi et al. 2011; Stock et al. 2012).

Anoxic environments are often characterized by sulfidic conditions, such as the Urania basin (Table 1). Because sulfide (as H_2_S or HS^−^) is highly toxic at low levels to most living organisms, eukaryotes thriving in anoxic, sulfidic environments need to detoxify sulfide. Even though not much is known about sulfide detoxification in protists, direct hydrogen sulfide consumption (Searcy 2006) and sulfide metabolism through symbiotic bacteria has been proposed for different lineages (e.g., Bernhard and Buck 2004; Edgcomb et al. 2011a). Therefore, sulfide acts as an additional factor that selects for different protists in anoxic and highly sulfidic environments (Orsi et al. 2011).

The variance in the observed protistan community patterns that is unaccounted for in our CCA may to some extent be attributed to biotic factors such as trophic interactions in the individual communities. Thus far, our knowledge of microbial webs in anoxic habitats (Massana and Pedrós-Alió 1994; Fenchel and Finlay 1995; Sacca et al. 2009) and in the deep sea is limited. Further efforts will be needed in order to reveal the biotic factors that influence the structure of protistan communities in the DHABs in the Eastern Mediterranean Sea. This T-RFLP analysis of water samples from five DHABs (Discovery, Urania, Thetis, Tyro, and Medee) allows us to understand that selective pressures drive the structure of protistan communities in deep-sea hypersaline anoxic basins. Beta-diversity between brine waters and haloclines with varying hydrochemistry appears to reflect widely divergent communities of microbial eukaryotes. Gradients of sodium, magnesium, sulfate, and oxygen within the haloclines appear to act as barriers to protistan dispersal, facilitating selection and diversification of unique communities.

## Supporting Information

**Figure S1.** Hierarchical clustering (Chao–Sørensen distance) of brine (B) and halocline interfaces (I) sampling sites based on T-RF size and relative abundance. D, Discovery; M, Medee; T, Tyro; Th, Thetis; U, Urania.
