# Supplementary figures and images for: Environmental selection of protistan plankton communities in hypersaline anoxic deep-sea basins, Eastern Mediterranean Sea
Source: Microbiologyopen. 2012 Dec 13;2(1):54–63. doi: 10.1002/mbo3.56 (PMC3584213; doi:10.1002/mbo3.56)

Suppl. Fig. 1

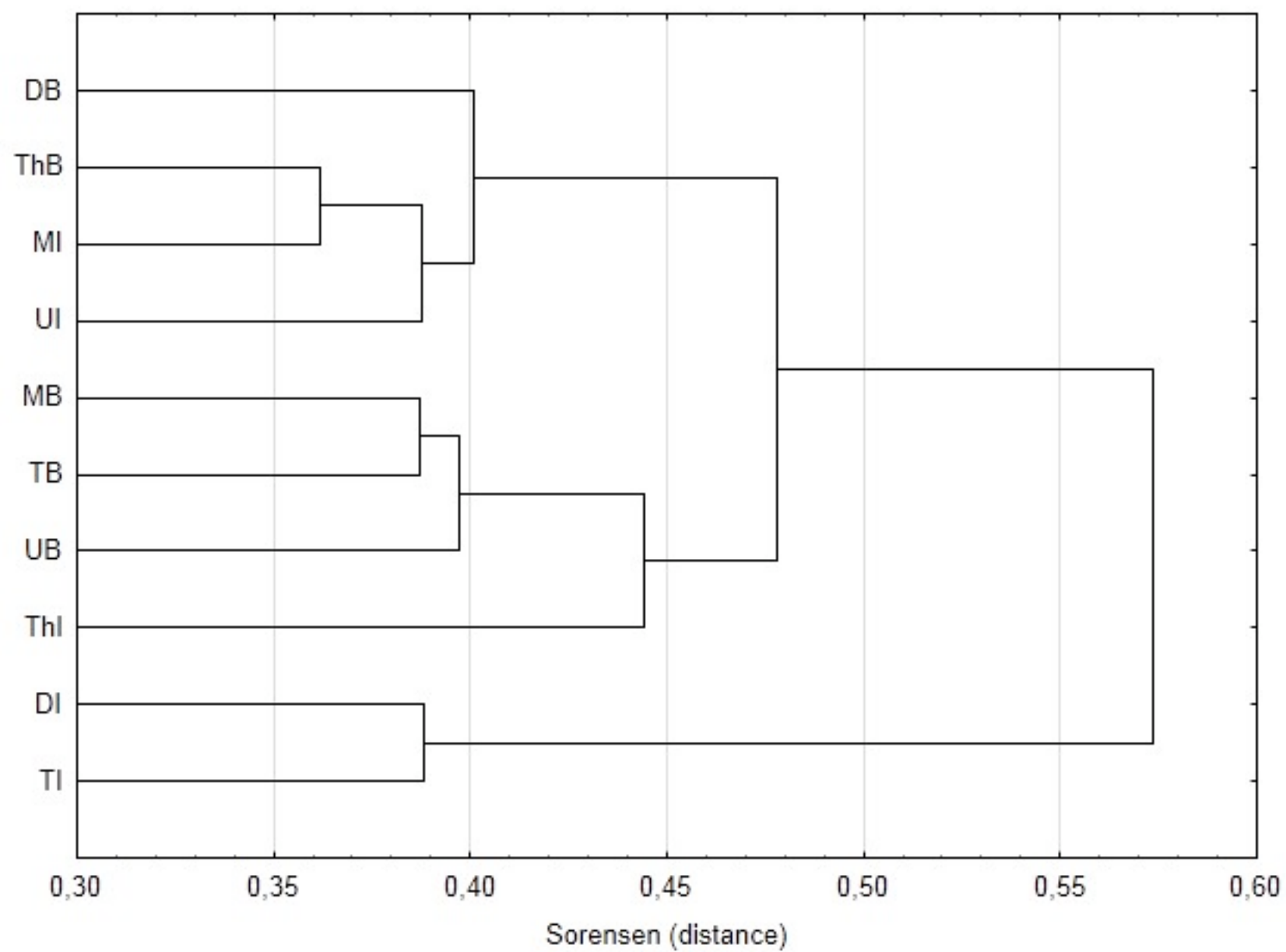

Supplement: Supplementary file 1 [file mbo30002-0054-SD1.pdf]
